# Effect of COVID-19 Pandemic Response and Parental Adverse Childhood Experiences on Child Health and Well-Being

**DOI:** 10.1007/s40653-023-00517-1

**Published:** 2023-02-13

**Authors:** Tolu Arowolo, Adeola Animasahun, Kesha Baptiste-Roberts, Yvonne Bronner

**Affiliations:** Department of Public Health, School of Community Health & Policy, Morgan State University, 4530 Portage Ave Campus, Ste 211 1700 E Cold Spring Lane, 21251 Baltimore, MD USA

**Keywords:** COVID-19, Parental adverse childhood experiences, Child well-being, Child health

## Abstract

Family responses to crises such as COVID-19 are driven by parents’ experiences. Parental history of adverse childhood experiences (ACEs) might play an important role in predicting resilience, coping capacity, and parenting practices during the COVID-19 pandemic response. The purpose of this review is to examine the impact of COVID-19 pandemic disruption on child health and well-being as influenced by the previous history of ACEs in the parents. Scopus, Google Scholar, PubMed, and PsychInfo were searched for peer-reviewed articles using the keywords “COVID-19”, “Parents or Maternal Adverse Childhood Experiences”, and “child health” or “child well-being”. Data were extracted using a literature review matrix template. Title, abstract, and full article-level reviews were conducted by two reviewers. The association between COVID-19 disruption, negative parenting, and child behavioral and emotional problems was stronger for parents with younger children with a history of high ACE scores. Parents with high ACE scores were more likely to cope poorly with childcare duties and engage in child neglect, verbal abuse, and reduced feeding frequency, specifically during the COVID-19 pandemic. The review findings support the framework of inadequate resilience and coping skills of adults with a history of ACEs during periods of stress and unpredictability such as the COVID-19 pandemic. The negative effects of these parental stressors on a child’s health and well-being are modifiable and could be mitigated by targeted interventions. Trauma-informed care should be adopted to contribute to optimum child health.

## Introduction

In the Spring of 2020, the World Health Organization declared the Coronavirus-19 disease a pandemic (“WHO Coronavirus Disease (COVID-19) Dashboard.,” 2020). Physical distancing measures were implemented globally as there was no vaccine against COVID-19 during the initial pandemic response phase. Travel restrictions were imposed, a stay-at-home order was enforced, schools were closed, and non-essential services were shut down. These measures led to social and economic disruption at individual, family, and community levels. The immediate effects of these initial responses to the COVID-19 pandemic were evidenced by massive job loss or reduction in the number of work hours, economic instability, housing issues, and food insecurity (Fisher et al., 2020). The profound effect of the immediate COVID-19 pandemic response in the context of family living has been described (Gröndal et al., 2021; Prime et al., 2020; Vatavali et al., 2020). Vulnerable families, especially those with low-income, unstable, or crowded housing, and limited employment flexibility faced a disproportionately higher risk of COVID-19 (Cluver et al., 2020; Gröndal et al., 2021; Prime et al., 2020; Vatavali et al., 2020). Some parents were faced with the problem of combining work from home and childcare, and these challenges were exacerbated for those living in crowded households (Cluver et al., 2020).

Stress associated with the COVID-19 pandemic, like any other period of unpredictability, has been documented to have a profound effect on everyday family life. Stress of this nature fuels parental stress and intrafamilial tension, causing an increase in lingering negative effects of past exposure to adverse childhood experiences (ACEs), including domestic violence, child abuse, and neglect (Fegert et al., 2020). ACE items from the original Kaiser Permanente ACE study (Felitti et al., 1998,) and the Philadelphia ACE study (Cronholm et al., 2015) include issues such as being a victim of bullying, experiencing foster care, exposure to domestic violence, and child neglect. Individuals with a significant history of exposure to ACEs have been reported to cope poorly during periods of unpredictability and stress, such as those posed by the COVID-19 pandemic (Daníelsdóttir et al., 2022; Hammen et al., 2000; Smid et al., 2012). The role of the caregiver’s well-being in supporting healthy parenting is critical. Parents who experienced ACEs may have challenges coping during periods of significant stress such as those posed by COVID-19 and may utilize unhealthy coping strategies (Boullier et al., 2018; Lange et al., 2019; Lê-Scherban et al., 2018).

**Fig. 1:**
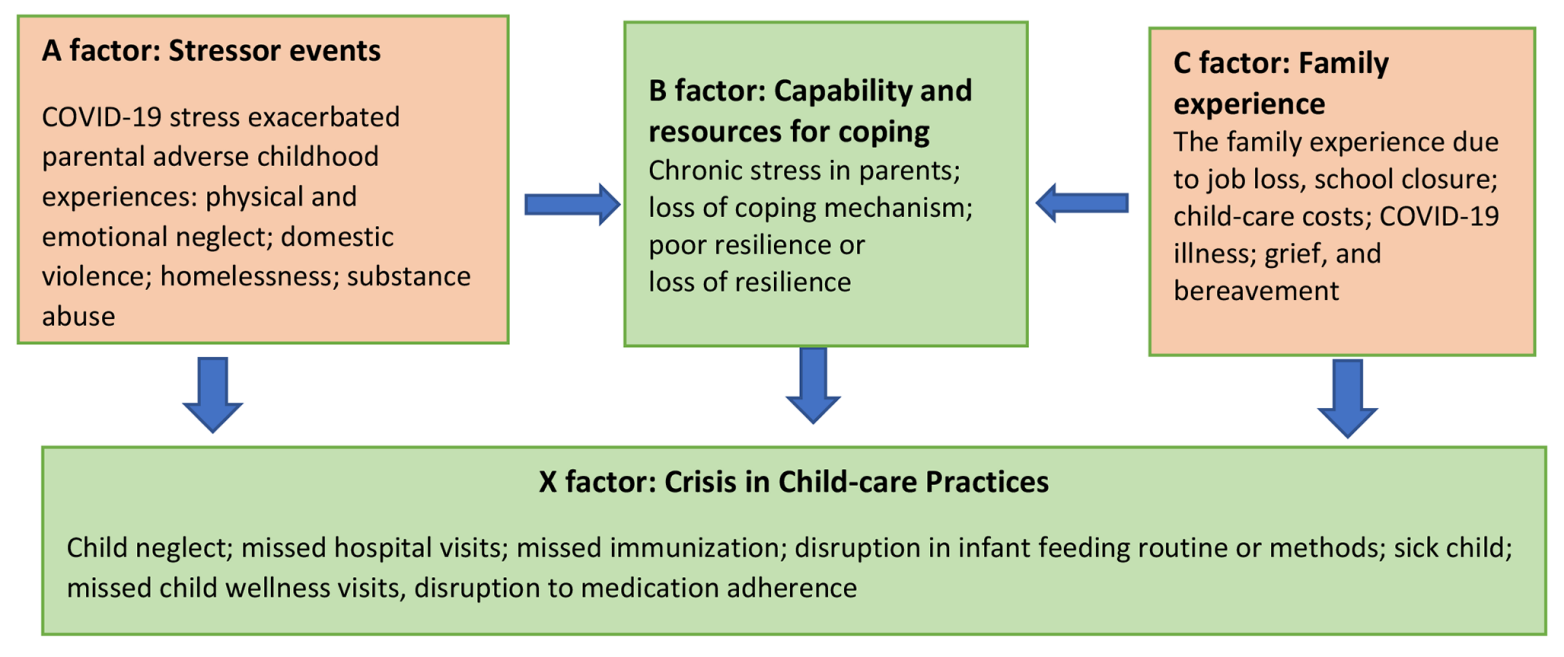
ABCX Family stress model (Hill, 1949) applied to the effect of the COVID-19 pandemic on child well-being influenced by parental ACEs.

The ABCX family crisis model highlighted that a family stressor, such as COVID-19 disruption, may impact the family functioning system (Patterson, 2002; Smith, 1984). Prior studies examined a link between an individual’s exposure to ACEs and their parenting stress and resilience during periods of stress and conflicts (Steele et al., 2016; Panisch et al., 2020). Figure 1 illustrates the hypothesized ABCX family stress model for COVID-19 family experience, parental history of ACEs, the consequent effect on childcare, and the subsequent impact on child health. COVID-19 stressors exacerbated by parental history of ACEs such as physical and emotional neglect, domestic violence, and substance use could be heightened by family experiences due to COVID-19 response. Further, according to the model, the resulting loss in capacity and resources for resilience or coping strategies could lead to increased stress in parents. According to research, the consequent effect on childcare practices may include child neglect, missed preventive visits (Lebrun-Harris et al., 2022), missed immunization opportunities, disruption in infant feeding routine or choices (Pacheco et al., 2021), missed outpatient visits and follow-up for sick children, and disruption to medication adherence (Teasdale et al., 2021). Providers’ lack of consideration of factors such as parents’ vulnerabilities or previous history of ACEs could limit interventions to address child well-being, including prevention of child maltreatment and promoting positive child-care practices such as immunization visits and medication adherence. This review intends to investigate whether having a parent with a history of ACEs disproportionately affects the impact of COVID-19 on the health and well-being of their children as predicted in the model in Fig. 1.

### Research Questions

How do previous parental ACEs impact child health and well-being during the COVID-19 pandemic disruption? Are parents with high ACEs scores more likely to struggle with coping compared to parents with low or no ACEs? Do parents with high ACEs exhibit negative parenting, including child maltreatment during the COVID-19 pandemic compared with parents with low or no ACEs?

## Methods

We adopted the framework outlined by Arksey and O’Malley (Arksey & O’Malley, 2005) for conducting a scoping review. PubMed, PsychInfo, Scopus, and Google Scholar were searched for articles published in English from January 1, 2020, to March 15, 2022, using the following keywords, “COVID-19”, AND “Parents OR Maternal Adverse Childhood Experiences”, AND “child health” OR “child well-being”. The Preferred Reporting Items for Systematic Review and Meta-analysis (PRISMA) flow chart was used to obtain, sequence, and summarize article identification, screening, and selection (Fig. 2). Articles were included if published within the time frame identified and if they primarily assessed whether the maternal or parental history of ACEs influenced child health and well-being during the COVID-19 pandemic time. Articles that described the overall impact of COVID-19 as moderated by parents’ or mothers’ previous history of ACEs or trauma adversity on child health were included. Articles that assessed the overall impact of COVID-19 on child health without assessing the effect of parental or maternal ACEs were excluded. Case reports, qualitative studies, observational studies, and systematic reviews were assessed for inclusion. Duplicates were manually removed. Full texts of all included articles were obtained for review.

**Figure:**
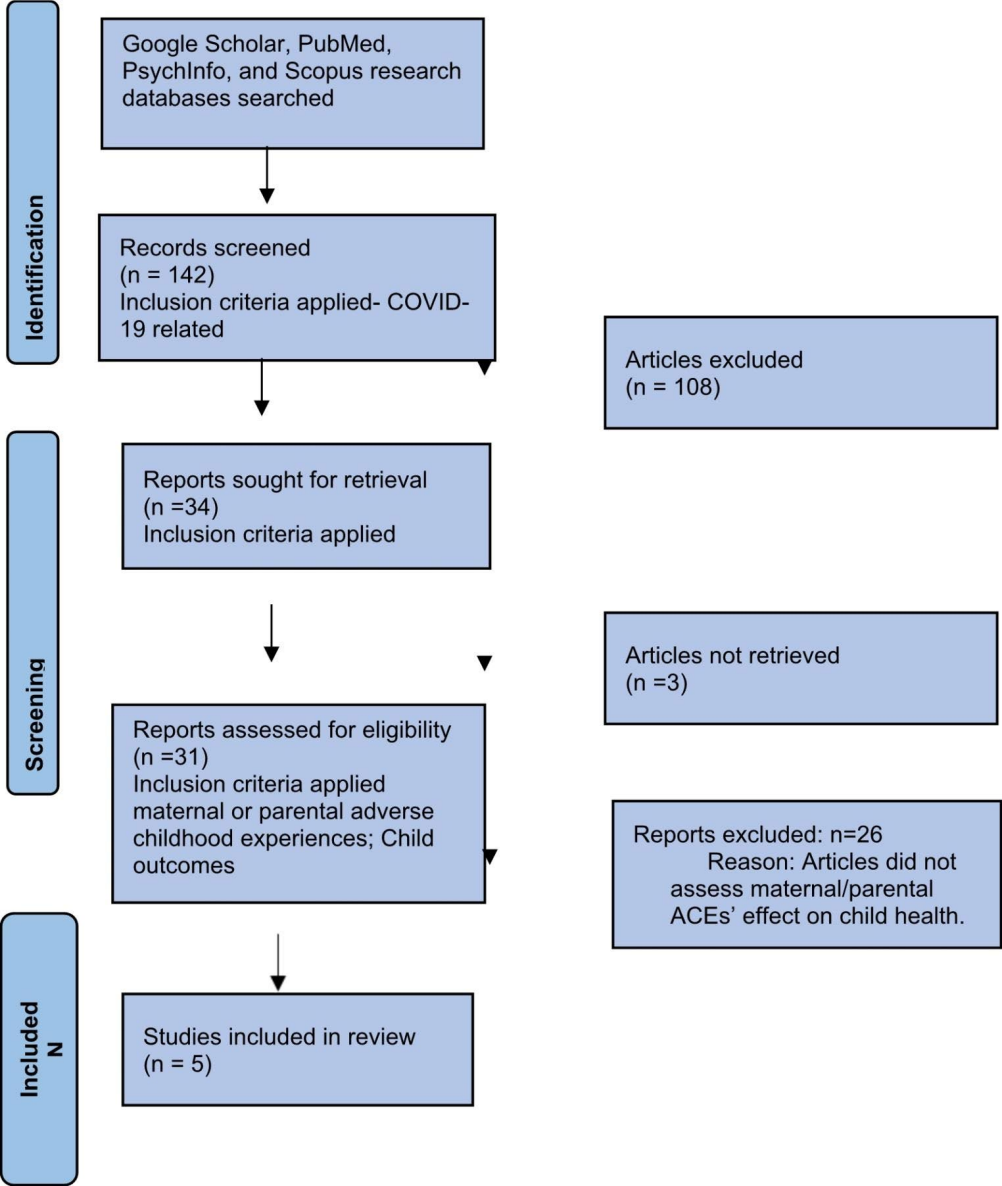
Figure 2

One hundred and forty-two (142) peer-reviewed articles were obtained in the original search. One hundred and eight (108) articles, after the abstract level review, were excluded due to the application of inclusion and exclusion criteria following the PRISMA flow. Full publications for thirty-four (34) articles were sought. Three (3) articles could not be retrieved, and an additional twenty-six (26) articles were excluded following individual full article reviews because they did not specifically assess the effect of maternal or parental ACEs on child health. Five (5) articles were included in the final review. Articles in the final review included four cross-sectional and one clinical cohort methodology. Two reviewers developed a literature review matrix for article and data abstraction. The specific items included the title of the articles selected, the study design or type of article, the study population characteristics, and key findings. Themes were identified from the key findings as abstracted by two reviewers. Themes were supported by all reviewers based on results obtained during abstraction. Narrative descriptions of the evidence were summarized for each theme and revised by all reviewers for clarity and relevance.

## Results

Five articles were included in the final review. Four (4) were cross-sectional in design and one (1) was a clinical cohort study. A total of 2,329 respondents comprising 73.5% of mothers with under-age children participated in the five studies. The remaining respondents were fathers (23.6%) and other caregivers (2.9%) including grandparents. Two of the studies were conducted in Germany, and the remaining were in the United States. Most of the study participants identified as White. The study population characteristics are summarized in Table 1.

**Table 1:** Summary of reviewed articles’ study design, population and sample characteristics, and key findings

Article/Title	Study design	Population /Sample characteristics	Scale/Instrument for variables studied	Summary of key findings
(Calvano et al., 2021) Families in the COVID-19 pandemic: parental stress, parent mental health and the occurrence of adverse childhood experiences-results of a representative survey in Germany.	Cross-sectional survey	97% White. 93% were middle- high-income earners 1,024 parents of minors in Germany Mothers: 50.9% Fathers: 44.7% Others: 4.3% Mean child age: 9.1 years (range 0.5–17)	Patient Health Questionnaire (PHQ) German Version Pandemic Stress Scale Parental Stress Scale Stress module of the PHQ (Cronbach’s alpha = 0.81) PHQ-4 for Parental mental health (Cronbach’s alpha = 0.86) Maltreatment and Abuse Chronology of Exposure (MACE) scale- ACE variables	Parental stress increased significantly during the pandemic. These families were characterized by higher parenting stress, job losses, young parent, and young child age. Parents with significant ACE scores reported negative coping patterns. Mothers scored higher than fathers on general stress (*d = 0.32, p < 0.001*), anxiety (*d = 0.28, p < 0.001*), and depression (*d = 0.28, p < 0.001*). Parental stress emerged as an important target point for interventions addressing the negative consequence of COVID-19. Parents of children younger than 5 years with a previous history of ACEs were more likely to report pandemic-related stress; *p = 0.014* and poorer parental outcomes.
(Clemens et al., 2021) Predictors of parental coping during the COVID-19 pandemic: A survey in Germany	Cross-sectional survey	Predominantly White 67% have a high school diploma and higher 687 parents of minors in Germany Mothers: 89.5% Fathers:10.5%	German version of the ACEs questionnaire (Cronbach’s alpha = 0.76) Survey questionnaire for parenting behavior and satisfaction	Results showed that the younger age of children (*B=-0.06, p < 0.005*), income loss (*B=-0.51, p < 0.05*), dissatisfaction with sharing childcare duties (*B= -0.84, p < 0.001*), and parental ACEs were significantly associated with an increase in negative parenting behavior during the COVID-19 pandemic. Parental satisfaction with sharing of caregiving is an important factor for parental coping during the pandemic.
(Hails et al., 2022) COVID-19 distress, negative parenting, and child behavioral problems: The moderating role of parent adverse childhood experiences	Cross-sectional survey	58% White; 14% Black 39% of low-income earners 267 parents of children ages 1.5-5 years in Oregon, Ohio, and Kansas Mothers: 85% Fathers: 8%	ACEs Questionnaire to assess parents’ ACEs (Cronbach’s alpha) = 0.80 Distress Scale from COVID-19 exposure and Family Impact Survey- COVID-19 family distress Negative Parenting scale from the Multidimensional Assessment of Parenting Scale (MAPS) Cronbach alpha = 0.87 The Depression subscale of the Patient-Reported Outcomes Measurement Information Systems-29 (PROMIS-29)- parental depressive symptoms Cronbach alpha = 0.92 The Preschool Pediatric Symptoms Checklist (PPSC)-to measure child emotional and behavioral problems Cronbach alpha = 0.90	Single parent status was significantly associated with parental ACEs (*r = 0.13, p < 0.05*), COVID-19 family distress (*r = 0.13, p < 0.05*), and child emotional and behavioral problems (*r = 0.29, p < 0.05*). Parental depressive symptoms were significantly associated with parental ACEs (*r = 0.31, p < 0.05*), COVID-19 family distress (*r = 0.40, p < 0.05*), negative parenting *(r = 0.31, P < 0.05*), and child emotional and behavioral problems (*r = 0.40, p < 0.05*). Negative parenting significantly mediated the relationship between COVID-19 distress and child emotional/behavioral problems (*b = 0.35, p < 0.01)* Parents’ ACEs moderated the associations between COVID-19 distress, negative parenting, and child emotional and behavioral problems. The relationship is stronger with a higher parental ACE score. The overall model of the mediating effect of COVID-19 distress on parental ACEs, negative parenting, and child outcomes was statistically significant (*F = 24.69, p < 0.01*)
(LaBrenz et al., 2021) Maternal Adverse Childhood experience exposure and resilience during COVID-19	Cross-sectional survey	80% White. 68% were high-income earners >$80,000 per annum. 250 mothers of children ages 0–5 years in Texas, U. S	Parenting Assessment of Protective Factors (PAPF) scale to measure parental resilience, social connections, concrete support, social and emotional competence) Cronbach alpha = 0.93 Expanded ACEs Questionnaire Cronbach alpha = 0.81 Survey questionnaire to assess childcare issues during COVID-19	Problems with childcare arrangements during COVID-19 *(b= -0.15*), and maternal ACE scores greater than 5 *(b = 0.26*) were negatively associated with resilience measured by the PAPF score. The ability to adhere to social distancing *(b = 0.03*) was positively linked to protective factors and resilience. Mothers with childcare issues and ACE scores greater than 4 had lower social connection scores and lower parenting competence (*b=-0.27, p = 0.02*)
(Shreffler et al., 2021). Childhood adversity and perceived distress from the COVID-19 pandemic	Clinical cohort study	40% White, 28% Black, others Hispanic and Native Americans Mean education in years = 12 years Data of 101 racially diverse and low-income women from a clinical cohort study in the U. S.	Survey questionnaire about health, social, and economic impacts, perceived change in stress, and well-being	The average maternal ACE score was 3. Mothers who reported childhood adversity reported higher levels of distress and poor mental health due to the pandemic (*b = 0.08, p < 0.01*). Self-reported loneliness mediates the association between childhood adversity and increased maternal distress *(b = 0.05, p < 0.01)*
		2,329 total study participants comprising 73.5% mothers and 23.6% fathers. Others including grandparents 2.9%		

### Thematic Findings

#### Parents’ History of ACEs

There was variability in the measurement of ACEs between the studies. The parental ACE score was ranked from a score of 0 representing no history of ACEs to 10 representing high exposure following the original Kaiser Permanente study. ACE measures in the reviewed articles assessed the domains of child maltreatment (physical abuse, emotional abuse, sexual abuse, physical neglect, and emotional neglect) and household dysfunction (substance abuse, mental illness of a family member, and parent’s disappearance through divorce, separation, or incarceration). Only one study used an expanded ACE score which included community dysfunction domains, such as the experience of racial/ethnic discrimination, living in an unsafe neighborhood, and economic hardship experience (LaBrenz et al., 2021). The most common ACEs reported were the experience of verbal abuse, domestic violence, bullying, a history of spanking, living with someone who was mentally ill, having parents who were divorced or separated, and having parents who experienced job insecurity.

#### Parents’ History of ACEs and COVID-19 Pandemic Response

Parents with high ACEs scores > 5 indicating significant experience of childhood adversity reported increased stress and poorer mental health due to the COVID-19 pandemic. This effect was moderated by the experience of social isolation (Shreffler et al., 2021; Calvano et al., 2021), and the closure of daycare (Calvano et al., 2021) because of the COVID-19 pandemic response. Parents of children younger than 5 years with a previous history of ACEs were more likely to report pandemic-related stress; p = 0.014 (Calvano et al., 2021), p < 0.005 (Clemens et al., 2021).

#### Resilience and Coping of Parents During the COVID-19 Pandemic

All the reviewed articles assessed the resilience and coping capacity of parents during the COVID-19 pandemic. Parenting Assessment and Protective Factor (PAPF) measures included parental resilience, social connections, supporting systems in times of need, and social-emotional competence. Inadequate social-emotional competence was reported commonly as parental stress during the early COVID-19 pandemic period (Calvano et al., 2021; Hails et al., 2022), and the supporting system indicated decreased parental satisfaction with sharing childcare duties, p < 0.001(Clemens et al., 2021) or reported issues with childcare frequency, p = 0.05 (LaBrenz et al., 2021). Parents with a higher ACE score of > 5 had lower scores on parental resilience compared with parents with no history of ACEs, p < 0.1 (LaBrenz et al., 2021; Calvano et al., 2021). Overall, parents with a high ACE score > 5 had lower average parenting competence scores than mothers with an ACE score of 0. Covariates of inadequate parenting competence commonly reported in the studies were migrant background, job loss during the pandemic, food insecurity, housing instability, and low socioeconomic status (Clemens et al., 2021; LaBrenz et al., 2021). The overall average PAPF was 2.41 in one of the studies indicating strong resilience based on the assessment of parental resilience measures. Respondents in this study were mainly White mothers with annual household incomes greater than $80,000 or higher (LaBrenz et al., 2021). Maintenance of family routine, parental satisfaction with sharing childcare duties, having a secured job, and the ability to maintain social distancing were identified as protective coping strategies (Clemens et al., 2021; LaBrenz et al., 2021).

#### Parents’ ACEs and Parenting Outcomes

Parents with anxiety and depressive symptoms due to COVID-19 distress, high ACE scores > 5, parenting stress associated with job loss (Clemens et al., 2021; Hails et al., 2022), and younger child age (Clemens et al., 2021; LaBrenz et al., 2021), were considered at high risk of potential harmful parenting behavior during the COVID-19 pandemic. Parents’ ACEs moderated the association between COVID-19 distress and negative parenting (Clemens et al., 2021; LaBrenz et al., 2021). Parents with a high ACE score > 5 have an increased risk of negative parenting with a dose-response relationship observed in that higher parents’ ACE scores were associated with an increased risk of negative parenting (Hails et al., 2022).

#### Parenting Outcomes and Impact on Child Health

Parental depressive symptoms due to COVID-19 were significantly associated with a history of ACEs and child emotional and behavioral problems, such as aggression, hyperactivity, conduct problems, anxiety, and depression (Hails et al., 2022). Parental depressive illness and history of maltreatment were identified as predictors of child maltreatment. Key childcare practices identified include disruption in childcare arrangements, and dissatisfaction with the frequency of childcare duties, such as feeding frequency (Clemens et al., 2021; LaBrenz et al., 2021). Only one study tested the moderating influence of parent ACEs on the mediating effect of negative parenting in the relationship between COVID-19 and child behavioral problems. The impact of negative parenting and parents’ ACE-moderated COVID-19 stress was greater among children from low-income families, and parents’ history of ACEs moderated the relationship between COVID-19 distress and child health outcomes (Hails et al., 2022).

## Discussion and Implications for Future Research

Despite the plethora of literature on the evidence of inadequate coping of individuals with a history of significant ACEs during the period of stress and unpredictability such as posed by the COVID-19 pandemic(Panisch et al., 2020; Patterson, 2002; Steele et al., 2016), few articles met the inclusion criteria for this review. This could indicate that little attention was given to how the history of trauma adversity in parents plays out in their children during the COVID-19 pandemic. The population characteristics in the reviewed articles were predominantly White families, indicating a paucity of data to fully describe the effect of parental ACEs on child health outcomes among racial minorities during the initial phase of the COVID-19 pandemic response. Studies have shown that ACEs are disproportionately prevalent among groups that have been historically oppressed and racially marginalized (Andrews et al., 2015; Hunt et al., 2017; Sonu et al., 2021). Compared to other racial groups, black parents were more likely to be employed as essential workers in low-income jobs, experienced job loss if employed in less secure employment, experienced issues handling childcare because of school closure, and lived in households where quarantine was less practicable (Mann et al., 2020). Individuals who reported considerable adversity in early life were likely to be unemployed or experiencing job instability, have considerable low income, did not complete high school (Metzler et al., 2017), suffered housing instability or homelessness (Montgomery et al., 2013), and food insecurity (Chilton et al., 2015). Future research should consider other racial and ethnic minorities to further understand the association between parents’ history of ACEs and child health during the COVID-19 pandemic period. This is necessary to determine culturally sensitive interventions specific to minority populations.

The findings in this review support the documented hypothesis that individuals who experienced significant ACEs coped poorly in times of stress and unpredictability (Doom et al., 2016; Nurius et al., 2015) with a consequent impact on childcare practices, and ultimately on child health and well-being (Power, 2020; (Cronholm et al., 2015; Steele et al., 2016). The responsibility for the increased frequency of childcare falls upon primary caregivers, especially mothers as indicated in this review. Most working mothers would experience work disruptions and parental stress related to childcare including feeding, hospital visits, medication administration, and routine care. This might increase the risk of physical and psychological abuse and child neglect. Dissatisfaction expressed with increased frequency of childcare duties and inadequate support from spouses or significant others could lead to child neglect, verbal abuse, and maltreatment. Unstable childcare arrangements are a risk factor for child maltreatment (Ha et al., 2015).

Parents, especially mothers, with significant ACEs history could benefit from interventions to mitigate the effect of parenting frustrations and stress on childcare practices, coping mechanisms, and symptoms of mental health that limit the capacity for effective parenting. Re-traumatic experiences, such as domestic violence and other forms of intrafamilial tension, were documented to have increased during the stay-at-home phase of the COVID-19 pandemic (Anurudran et al., 2020; Kourti et al., 2021), acting to increase the risk of parenting stress and consequent negative parenting outcomes. Future research is required to assess ways to ameliorate negative parenting patterns among caregivers with significant ACEs, including as it pertains to caring for children during COVID-19. A trauma-informed approach is required as a framework to guide the understanding of providers about the effects of trauma, identify the signs, and respond to individuals under their care including children during COVID-19 disruptions. Assessing early trauma or adversity may help to identify parents and caregivers who would require additional support or interventions for positive parenting during a crisis. Maternal and child health programs should consider the integration of ACE screening and trauma-informed care as part of the standard of care for pre-and post-natal services during periods of family disruptions. Specific interventions based on trauma-informed care could also be integrated into the post-natal Doula program for new mothers.

Child maltreatment is a very important ACE issue. It is both a predictor and a consequence of adverse childhood experiences. Parents who experienced child maltreatment, such as verbal and domestic abuse or child neglect, are likely to maltreat their children (Michl-Petzing, 2018; Michl-Petzing et al., 2019; Savage et al., 2019; Widom et al., 2015). As the pandemic comes under control and COVID-19 vaccination is promoted to prevent future outbreaks, it is important to consider the impact of the pandemic response on children’s behavioral and physical health. Healthcare providers should be vigilant of the increased behavioral and mental health needs of children and adolescents after the pandemic. Due to significant missed cases of child abuse and neglect during the pandemic (Barboza et al., 2020; Rapoport et al., 2020), children with behavioral consequences of maltreatment could progress to severe psychological and developmental problems. This calls for extreme urgency among pediatricians, case workers, and other disciplines related to childcare in these COVID-19 times.

There are some limitations to this review. The reviewers consulted only four research databases and were unaware of unpublished studies on the topic. Only five (5) published studies addressed the research question, which is concerning, taking into consideration the attention that ACE studies have attracted in recent times. It is possible that at the time the review was conducted, researchers had not yet published their studies on the question, given the short time involved or some researchers might still be investigating the impact of COVID-19 on parental ACEs and child outcomes. Although the hypothesized effect on child outcomes was confirmed by the review findings, the review noted that most respondents in the reviewed articles are white and high-middle income earners. Future research should focus on collecting data from under-represented and low-income communities to validate these findings.

## Conclusion

This review finding supports the framework that parental ACEs play an important role in predicting the resilience and coping skills of parents, especially mothers, during periods of stress and unpredictability, such as the COVID-19 pandemic. Parents with significant ACEs history who reported having young children, limited support for childcare, and job loss have a high risk for negative parenting practices, such as child maltreatment, child neglect, changes in infant feeding options, missed immunization services, and hospital attendance. The impact of ACEs on parents with consequent effects on child health and well-being are modifiable and could be mitigated by targeted interventions, such as post-natal ACE screening and trauma-informed care. It is recommended that trauma-informed care could be adopted at the clinical and community level to address negative parenting practices that contribute to sub-optimum maternal and child health. Future research could consider the mediating role of parental ACEs on COVID-19 pandemic distress, parental outcomes, and the effect on child health among racial minorities and low-income families.
